# Analysis of repeated measures of multiple surgical sites within a factorial randomised controlled trial

**DOI:** 10.1186/1745-6215-16-S2-P126

**Published:** 2015-11-16

**Authors:** Gemma Clayton, Katie Pike, Gianni D Angelini, Chris A Rogers

**Affiliations:** 1 Clinical Trials and Evaluation Unit, School of Clinical Sciences, University of Bristol, Bristol, UK

## Background

In heart bypass surgery, pieces of artery or vein (“conduits”) taken from elsewhere in the patients’ body are used to bypass the blocked or narrowed coronary arteries and improve the blood supply to the heart. The “Hyperplasia and Atherogenesis in Bypass Vein Grafts Following Different Surgical Preparation Techniques” (HArVeST) trial, is a single-centre study investigating if new methods of taking and preparing the conduit can improve the outcome for patients.

## Design

The study has a 2x2 factorial design: (a) the method of taking the vein (pedicle harvest (leg vein and surrounding fat) versus conventional harvest (surrounding fat stripped from vein)) and (b) method for checking the vein is not damaged (low versus high pressure test).

The primary outcome is the patency of the vein graft(s) 12 months after surgery, i.e. thickness of the wall of the graft and size of the lumen, assessed using intravascular ultrasound (IVUS). Multiple measurements are taken as the IVUS machine moves along the graft. Baseline measurements are assessed from a histological analysis of vein harvested.

## Statistical analysis

Analyses of the co-primary outcomes, which are both continuous measures, are on-going. Mixed regression models with different correlation structures are being explored to account for correlation arising from: (a) multiple grafts and conduits from one patient, (b) grafts taken from the same conduit, and (c) the repeated measures nature of the IVUS measurements within a graft. Methodological considerations are multi-faceted; our aim is to make maximal use of the data within a hierarchical framework.

## Disclaimer

This research is supported by a National Institute for Health Research (NIHR) Bristol Cardiovascular Biomedical Research Unit. The views and opinions expressed therein are those of the authors and do not necessarily reflect those of the NIHR, NHS or the Department of Health.

